# Performance and Micro-Pacing Strategies in a Classic Cross-Country Skiing Sprint Race

**DOI:** 10.3389/fspor.2020.00077

**Published:** 2020-06-26

**Authors:** Simo Ihalainen, Steffi Colyer, Erik Andersson, Kerry McGawley

**Affiliations:** ^1^Swedish Winter Sports Research Centre, Department of Health Sciences, Mid Sweden University, Östersund, Sweden; ^2^Research Institute for Olympic Sports, Jyväskylä, Finland; ^3^Department for Health, University of Bath, Bath, United Kingdom

**Keywords:** competition, elite athletes, GNSS, GPS, statistical parametric mapping, time trial

## Abstract

The purpose of the present study was to analyze micro-pacing strategies in cross-country skiing, and their relation to section and total race times. Eleven competitive female cross-country skiers were tracked during a classic sprint time-trial race using a global navigation satellite system (GNSS) unit. A coordinate mapping procedure was applied to the GNSS unit measurements to analyze the instantaneous velocities and split times. The track was divided into nine sections and individual section times were calculated. Statistical parametric mapping (SPM) was used to analyze the relationships between instantaneous velocity and section or total race time. SPM analyses revealed two uphill sections and one flat section where greater instantaneous velocities were related to faster total race times. The first major uphill section on the track demonstrated a more conservative micro-pacing strategy (SPM supra-threshold clusters along the entire uphill, *p* < 0.05–0.001) compared to the more aggressive strategy used in the last uphill section (clusters on the first half of the uphill, *p* < 0.05–0.001). Faster flat section times were associated with greater instantaneous velocities throughout the section (*p* < 0.001), while faster downhill section times were related to greater instantaneous velocities at the top of the downhill (*p* < 0.001), and in the downhill turns (*p* < 0.001). In conclusion, micro-pacing strategies were related to overall skiing performance and distinct track sections were identified where instantaneous velocities were related to section or total race times. In order to improve skiing performance, athletes could focus on more aggressive pacing early on in the “end spurt”, during the transitions from uphill to flat sections, and during the transitions from flat or uphill to downhill sections.

## Introduction

Cross-country skiing is a physically demanding, whole-body endurance sport involving different competition distances and durations, course profiles, skiing speeds, and choices of sub-techniques (Sandbakk and Holmberg, 2017). All of these factors place unique challenges on the optimization of pacing strategies in cross-country skiing (Andersson et al., 2010, 2019; Sundström et al., 2013; Sandbakk et al., 2016).

Pacing strategies have been categorized in various sports as negative, positive, even, all-out, parabolic-shaped, and variable (Abbiss and Laursen, 2008). In cross-country skiing, athletes have been shown to use a positive pacing strategy when analyzed on a lap-to-lap basis (that is, lap times are faster at the start of a race and become subsequently slower) (Andersson et al., 2010; Bolger et al., 2015; Losnegard et al., 2016; Marsland et al., 2017). However, an analysis of elite male skiers' competition results showed that the faster athletes decreased their skiing velocity to a lesser degree than slower athletes and it was, therefore, suggested that the lesser-performing athletes may benefit from a more even pacing strategy (Losnegard et al., 2016).

In addition to lap-to-lap analyses, pacing strategies in cross-country skiing have been investigated over different track sections. Competitive cross-country skiing courses are comprised of approximately equal lengths of flat, uphill, and downhill sections. Previous studies analyzing section times, as well as velocities, have shown that performance in the uphill sections is the most important factor in determining overall performance (Andersson et al., 2010; Sandbakk et al., 2011, 2016; Bolger et al., 2015). For example, in a women's 10-km classic cross-country skiing time trial the total uphill time explained 95.5% of the variance in overall performance (Sandbakk et al., 2016). In addition, sex differences in sprint skiing performance have been shown to increase during uphill sections, while substantially lower differences between the sexes were reported on the faster flat and downhill course sections (Andersson et al., 2019). Flat sections have also been shown to relate to overall performance, whereas downhill sections have demonstrated both significant and non-significant correlations to overall performance (Andersson et al., 2010; Sandbakk et al., 2011, 2016; Bolger et al., 2015).

Due to the importance of uphill sections in determining overall skiing performance, it is not surprising that cross-country skiers demonstrate greater metabolic rates, produce more power, and accumulate greater oxygen deficits in uphill compared to flat sections (Andersson et al., 2016; Gløersen et al., 2018b; Karlsson et al., 2018). In fast downhill sections, skiers use a tucked position, produce no power (Swarén and Eriksson, 2017; Gløersen et al., 2018b), and are able to recover part of the previously accumulated oxygen deficit (Mognoni et al., 2001; Welde et al., 2003; Sandbakk and Holmberg, 2017; Gløersen et al., 2019). These differences in energy demands and power production during different track sections clearly indicate that a variable pacing strategy is utilized in cross-country skiing (Swarén and Eriksson, 2017; Gløersen et al., 2018b; Karlsson et al., 2018), rather than a strictly positive pacing strategy as suggested in studies using lap-to-lap analyses. Mathematical simulation studies in cross-country skiing and cycling have shown that a slight change in the power output profile, with an increase in power output on uphill sections and a decrease in power output on downhill and flat sections, can lead to improved performance, despite total metabolic power remaining the same (Swain, 1997; Sundström et al., 2013). This improved performance with an altered power output profile was attributed to a reduction in air drag resulting from a reduction in speed variation over the course (Sundström et al., 2013).

While section analyses have provided interesting insights into overall cross-country skiing performance, details about within-section pacing strategies (i.e., micro-pacing strategies) are lacking. That is, it is not currently known whether faster performances are related to faster skiing throughout a whole uphill or flat section compared to slower performances, or whether faster skiers outperform their slower counterparts in distinct parts of the track sections. Two previous studies have reported the propulsive power of skiers throughout different track sections, but differences between athletes or pacing strategies in relation to skiing performance were not investigated (Swarén and Eriksson, 2017; Gløersen et al., 2018b).

Recently, Gløersen et al. (2018a) showed that modern sports global navigation satellite system (GNSS) measurement units were able to accurately measure position and velocity over an entire cross-country skiing track, enabling instantaneous time and velocity analyses. The same measuring technology is also applicable during competition situations due to its low mass and negligible interference on the skier. From a performance perspective, it would be important to better understand how different track sections and transitions between sections in cross-country skiing should be paced. Therefore, the purpose of the present study was to analyze the micro-pacing strategies in a classic cross-country skiing sprint time trial and to examine how these micro-pacing strategies are related to section times and total race times.

## Materials and Methods

### Subjects

Eleven female cross-country skiers (age 23.9 ± 2.7 years, sprint FIS points 102 ± 24) competing in a specific Scandinavian cup classic sprint competition volunteered and gave their written consent to participate in the study. The participant group included athletes from three different countries and ski teams. The study was conducted according to the declaration of Helsinki, and ethical approval was granted by the regional ethical review board of Umeå University, Sweden (#2018-441-32M).

### GNSS Measurements, Equipment, and Weather Conditions

Participants were equipped with a GNSS and inertial sensor unit (OptimEye S5, Catapult Sports, Australia) immediately prior to the qualification round (prologue) of the sprint competition, which was conducted as a time trial (i.e., with individual starts). The unit was placed in the middle of the upper back in a standard vest provided by the manufacturer. Apart from the process of attaching the measurement unit, which took ~1 min, participants performed their individual warm-up and competition routines as normal. The skiers used their own racing skis, boots, and poles. The snow was fresh and fine-grained, and the ambient temperature was −12°C. Although ski waxing was not standardized, all skis were selected and prepared for prevailing snow conditions by professional ski technicians.

The competition sprint track was a single, 1,380-m lap with a maximum elevation difference of 29 m and a total climb of 52 m. The GNSS unit measured the athlete's position and velocity at 10 Hz throughout the qualification race. The data was stored internally in the measurement unit and downloaded after the competition onto a laptop for post-processing.

In order to apply a coordinate mapping procedure to the GNSS race data, a mapping trajectory was measured before the competition start using a differential GNSS (dGNSS) device (Leica Zeno GG04+, Leica Geosystems, Switzerland). The dGNSS device had a 1 cm ± 1 ppm horizontal and 2 cm ± 1 ppm vertical positional accuracy in the real-time kinematics mode that was used. The reference track was measured by placing the dGNSS device on a sledge, which was pulled around the official competition course by walking along the ski track. Start- and finish-line locations were measured separately by placing the dGNSS unit stationary at each side of the competition track for 30 s.

### Data Processing

In order to compare skiers' split times and velocities at the same track locations, and to improve the GNSS measurement accuracy, the location and velocity data were processed according to a coordinate mapping procedure used previously (Gløersen et al., 2018a,b). Firstly, the mapping trajectory coordinates were filtered with a Butterworth low pass filter with a 0.3-Hz cut-off frequency. The mapping trajectory coordinates were then re-sampled at 1-m intervals and interpolated using a cubic spline. Secondly, the GNSS race data (coordinates and velocity) were filtered with the same Butterworth filter. The GNSS coordinate data were then mapped onto a point along the mapping trajectory where the Euclidean distance between the measured location and the mapping trajectory was minimized. Lastly, in order to evaluate the split-time and velocity differences between athletes at identical positions along the mapping trajectory, the mapped GNSS data time-stamps and velocities were linearly interpolated to every integer meter along the mapping trajectory.

**Figure 1:**
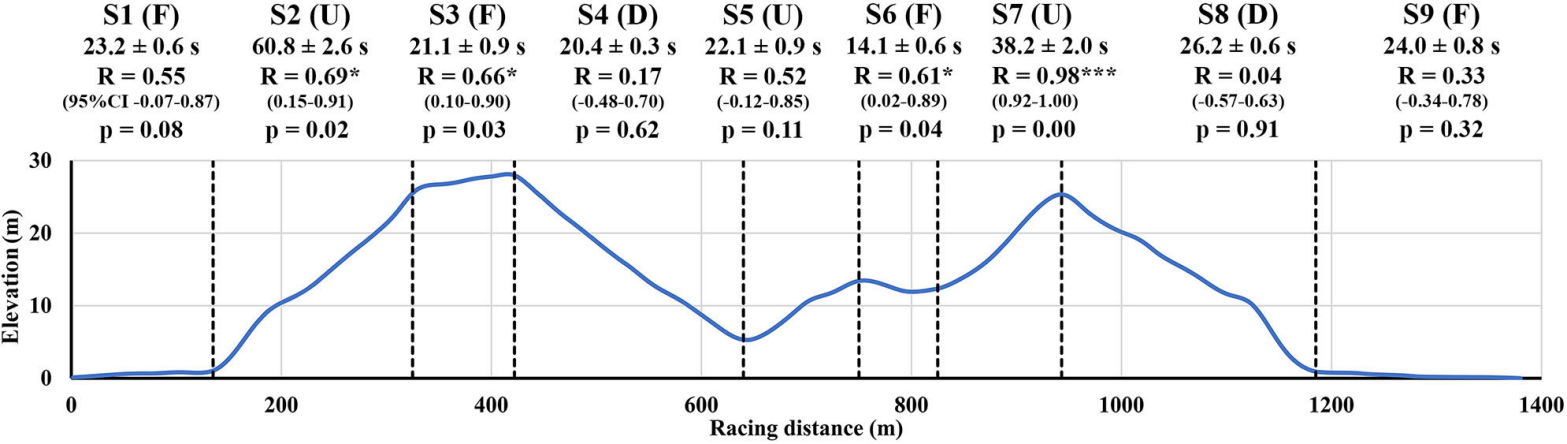
Uphill (U), flat (F), and downhill (D) track sections (S1–S9), with section times (s), correlation coefficients (R) for section time vs. total race time, and 95% confidence intervals (CI) for correlation coefficients.

The track was divided into nine sections consisting of three uphill (S2, S5, and S7), four flat (S1, S3, S6, and S9), and two downhill (S4 and S8) sections (Figure 1). The uphill sections had mean inclines of 7.4°, 4.2°, and 6.2°, respectively, while the flat sections had mean inclines of 0.4°, 1.3°, −0.8°, and −0.3° and the downhill sections had mean inclines of −5.9° and −5.7°, respectively. Section times were calculated from the mapped GNSS data for each section.

### Statistical Analysis

Two-tailed Pearson's correlation coefficients were computed to examine the relationship between section times and total race time. The instantaneous velocity curves (1-dimensional; 1D data) from all nine track sections (S1–S9) were analyzed using a statistical parametric mapping (SPM) procedure to analyze the track sections where instantaneous velocity was related to section or total race time (0-dimensional; 0D data). SPM enables the analysis of the whole 1D velocity curve rather than discrete 0D variables calculated from the velocity data and takes into account the smoothness of the 1D data. For each section, SPM 1D one-tailed linear regression models were applied resulting in SPM{t} curves. Where the SPM{t} values exceeded a critical threshold of α = 0.05 (i.e., that only 5% of random curves with the same smoothness would exceed the threshold), instantaneous velocity was considered to be significantly related to the section or total race time. Lastly, the probability that these supra-threshold regions could have been formed from equally smooth random curves was calculated.

Data are presented as mean ± standard deviation. Pearson's correlation coefficients were computed with IBM SPSS statistics 26 (IBM Co., Armonk, New York, USA) and the SPM analysis was conducted using open-source SPM 1D software (Pataky, 2012) in MATLAB R2018a (The MathWorks, Inc., Natick, Massachusetts, USA).

## Results

The total race time was 250.4 ± 5.8 s, which corresponded to a mean skiing velocity of 5.5 ± 0.1 m·s^−1^. Mean velocities for the uphill, flat, and downhill sections were 3.5 ± 0.1 m·s^−1^, 6.1 ± 0.1 m·s^−1^, and 9.9 ± 0.2 m·s^−1^, respectively. The uphill section times for S2 and S7 and flat section times for S3 and S6 were significantly correlated to total race time, whereas downhill section times (S4 and S8) showed no relation to total race time (Figure 1).

**Figure 2:**
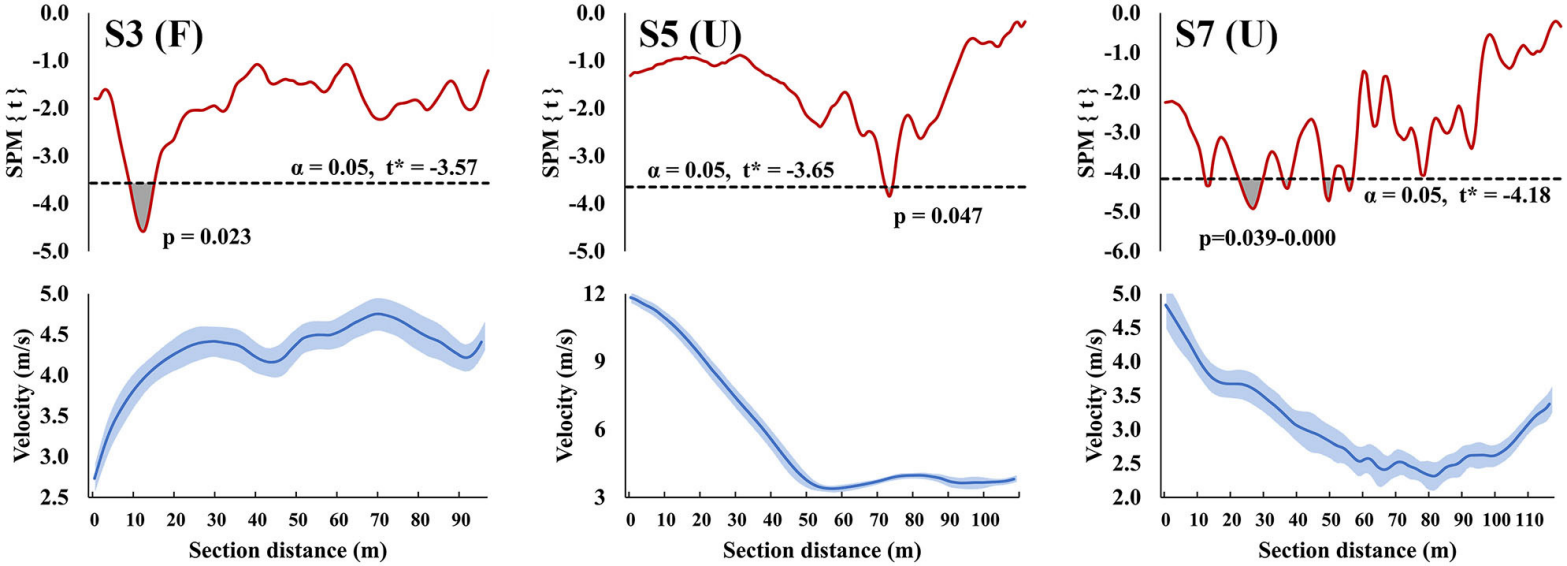
Statistical parametric mapping (SPM{t}, upper row) and instantaneous velocity (lower row) with mean (hard line) ± standard deviation (shaded area) curves from the flat (F) and uphill (U) track sections S3, S5, and S7 (i.e., those sections where instantaneous velocity was related to total race time). The shaded areas on the SPM{t} curve show the track locations where the SPM{t} curve exceeded the critical threshold (dotted line) and where statistically significant relationships between instantaneous velocity and total race time were present. *P*-values are provided for these supra-threshold clusters. The negative relationships presented indicate that, as expected, greater instantaneous velocities were related to faster total track times.

SPM regression analyses revealed three track sections (two uphill and one flat) where instantaneous velocity was related to total race time (Figure 2). On the flat section (S3), faster total race times were related to higher velocities during the 10 to 15-m distance immediately following the preceding uphill. Over this distance the mean skiing velocity increased from 3.7 ± 0.2 m·s^−1^ to 4.1 ± 0.2 m·s^−1^. In the last uphill on the track (S7), faster total race times were related to higher velocities at five track locations during the first half of the uphill (i.e., at 10–55 m). Over this distance the mean skiing time was 15.0 ± 1.0 s and mean skiing velocity decreased from 3.9 ± 0.2 m·s^−1^ to 2.7 ± 0.2 m·s^−1^. The fastest athlete was able to gain 3.2 s against the slowest athlete over this distance.

**Figure 3:**
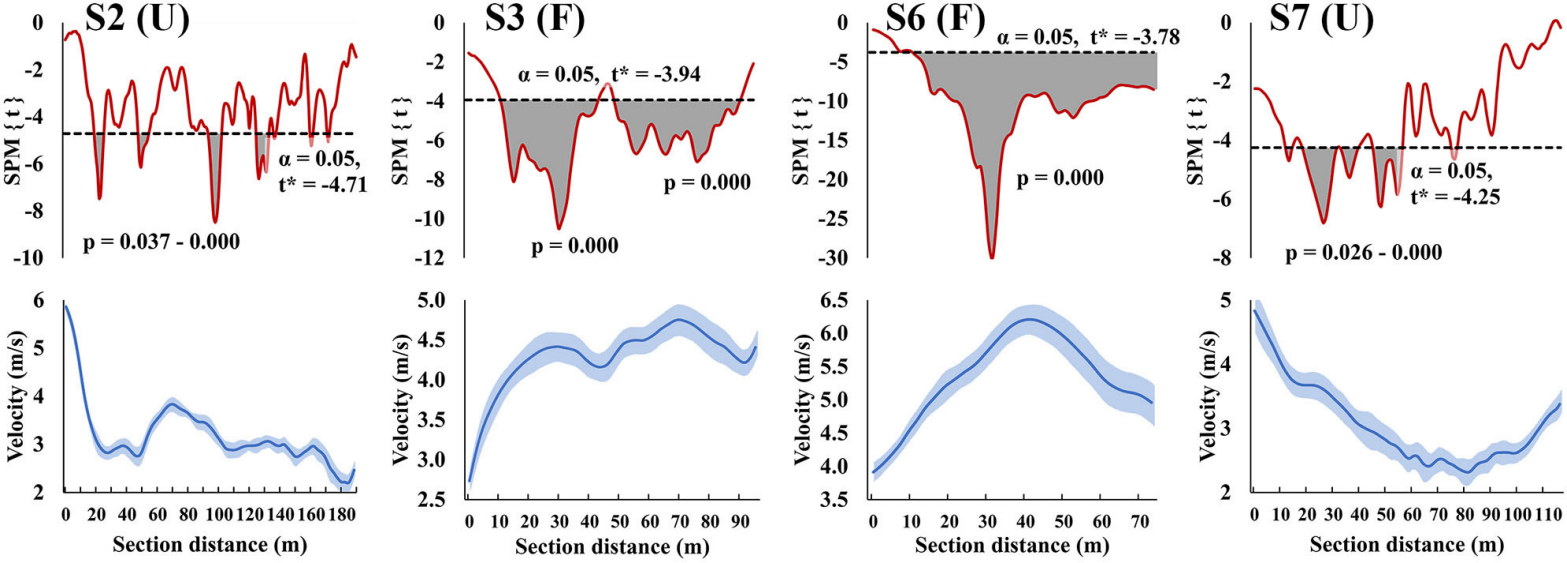
Statistical parametric mapping (SPM{t}, upper row) and instantaneous velocity (lower row) with mean (hard line) ± standard deviation (shaded area) curves from the uphill (U) and flat (F) track sections S2, S3, S6, and S7 (i.e., those sections where section time was related to total race time). The shaded areas on the SPM{t} curves show the track locations where a statistically significant relationship between instantaneous velocity and section time was present. *P*-values are provided for these supra-threshold clusters. The negative relationships indicate that greater instantaneous velocities were related to faster section times.

The SPM regressions between instantaneous velocities and section times are presented in Figure 3 for the four sections (uphill track sections S2 and S7; flat track sections S3 and S6) where section time was related to total race time. The SPM regressions showed different patterns in the relationships between instantaneous velocity and section times for the four different track sections. During the first uphill (S2), better section times were related to higher instantaneous velocities on several occasions throughout the whole uphill section. In contrast, better section times in the last uphill (S7) were related to higher instantaneous velocities during the first half of the uphill. On the flat sections (S3 and S6), better section times were related to higher instantaneous velocities almost throughout the whole section. The exception on both of these flat sections was the initial acceleration phase (at 0–12 m) after the preceding uphill, where skiing velocity increased from 2.7 ± 0.2 m·s^−1^ to 4.0 ± 0.2 m·s^−1^ in S3 and from 3.9 ± 0.2 m·s^−1^ to 4.7 ± 0.2 m·s^−1^ in S6. Only after this initial acceleration phase was the instantaneous velocity related to the section time and the fastest athletes were able to reach higher velocities from 12 m onwards (maximal skiing velocity: 4.8 ± 0.2 m·s^−1^ on S3 and 6.2 ± 0.2 m·s^−1^ on S6).

**Figure 4:**
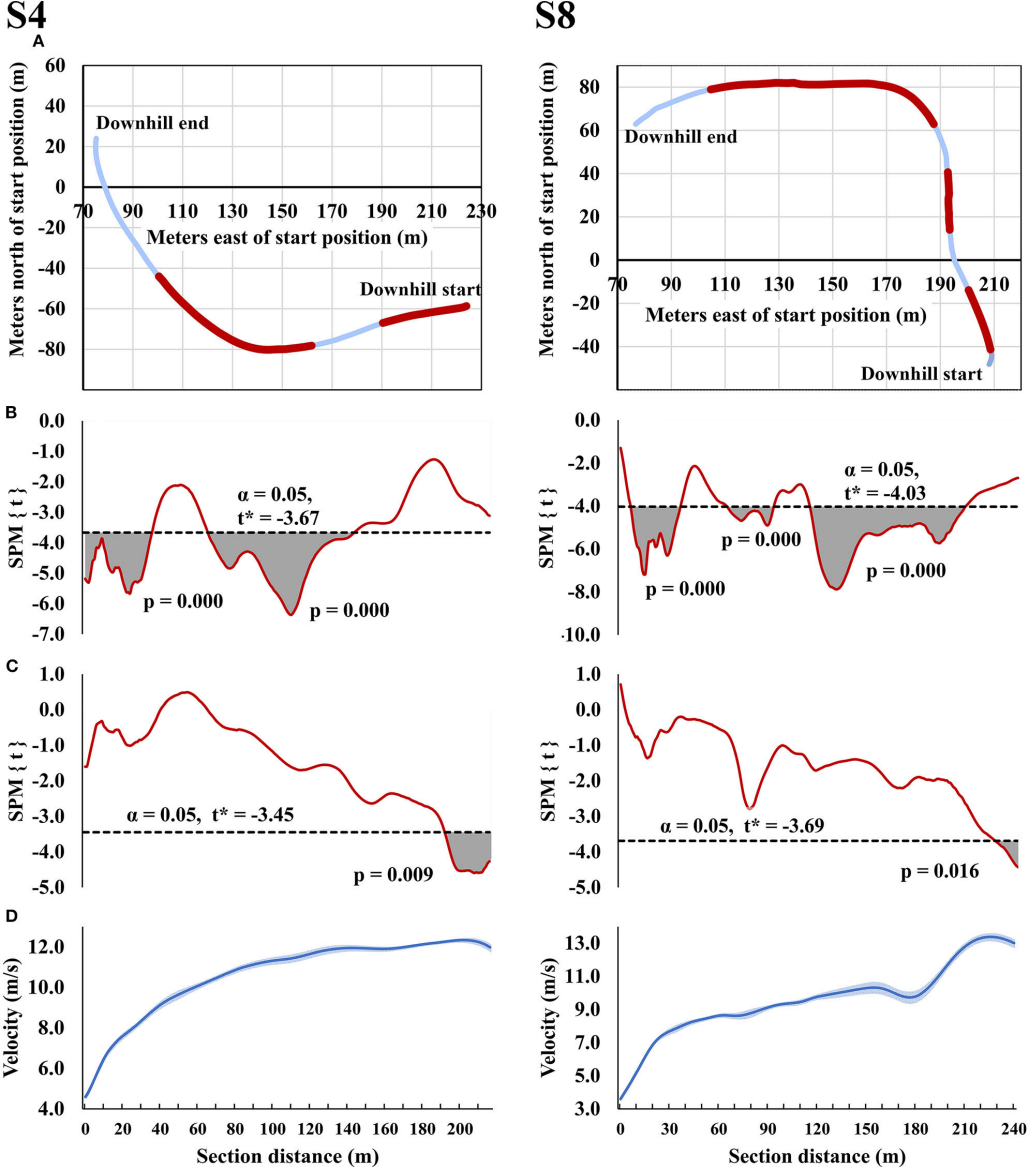
Downhill sections S4 and S8 showing **(A)** horizontal trajectories with highlighted regions where statistically significant relationships between instantaneous velocity and section time are present, **(B)** statistical parametric mapping (SPM{t}) curve showing the statistically significant relationships between instantaneous velocity and section time, **(C)** SPM{t} curve showing the statistically significant relationships between instantaneous velocity and the section time in the subsequent section following the downhill, and **(D)** instantaneous velocity in the downhill.

Both downhill sections (S4 and S8) demonstrated similar relationships between the section times and instantaneous velocities in the initial acceleration part of the downhill and the downhill turns (Figure 4). In these parts of the downhill, better section times were related to higher instantaneous velocities, whereas the highest velocities reached at the end of the downhills were not related to the downhill section times. However, the highest velocities at the end of the downhills were related to the subsequent section times (i.e., S5 and S9, respectively).

## Discussion

The results of the present study have shown that in a classic cross-country skiing sprint time trial, athletes used different micro-pacing (i.e., within-section pacing) strategies on the two major uphill sections on the track. A more conservative micro-pacing strategy was used in the first uphill, where the instantaneous velocities were not related to total race time. However, faster total race times were related to greater instantaneous velocities in the initial part of the subsequent flat section straight after the uphill. A more aggressive micro-pacing strategy was used in the last uphill on the track, where the instantaneous velocities during the first half of the climb were related to total race time. Faster times recorded on the flat sections were associated with greater instantaneous velocities throughout the section, while faster times recorded on the downhill sections were associated with greater instantaneous velocities during the acceleration phase at the top of the downhill, as well as during the downhill turns.

Previously, uphill sections in cross-country skiing have been shown to be the most important sections in determining overall performance (Andersson et al., 2010; Sandbakk et al., 2011, 2016; Bolger et al., 2015). The results of the present study support these findings and further describe uphill micro-pacing strategies and their relation to section and total race times, with different patterns observed on different uphill sections. The first uphill was characterized by more even pacing, where better section times were related to greater instantaneous velocities throughout the uphill. However, the micro-pacing strategies in this first uphill were not related to total race time. The first uphill was followed by a flat section, where higher velocities in the initial part of the section were related to total race time. Previously Karlsson et al. (2018) have stated that pacing in cross-country skiing could be affected by the length and incline of the uphill section, the position of the section in relation to the total track length, and the perceived difficulty of the subsequent sections ahead. All of these factors could have led to the more conservative micro-pacing strategy used in the first relatively long (~60 s) uphill. The results of the first uphill also highlight the importance of the transition from the uphill to the flat section. It might be that this type of track section ought to be paced such that the skier has enough remaining energetic resources at the top of the uphill to accelerate and reach a high velocity in the initial part of the subsequent flat section, in order to gain time against other competitors.

The final uphill on the track showed that better section times and overall performances required the athletes to ski faster in the first half of the climb. This uphill was followed by a long downhill and a relatively short finish straight, so it might be that the athletes did not have to save energetic resources for the subsequent sections, and in order to go all out on the last uphill had to pace more aggressively from the beginning of the uphill. This type of “end spurt” has been previously described in self-paced trials, where power output is increased in the final stages due to the reduced uncertainty regarding the remaining work (Tucker, 2009). Anaerobic capacity and maximal accumulated O_2_ deficit have also been related to cross-country skiing sprint performance (Losnegard et al., 2012; Andersson et al., 2016, 2017), which may enable better performing skiers to ski faster in the final uphill. Differences in micro-pacing strategies adopted in major uphill sections of any track might be affected by the preceding and subsequent sections of the track, and the placement of the section in relation to the total track length. Therefore, additional studies using different track profiles and track lengths are needed to confirm the differences in micro-pacing strategies for different types of uphill sections.

As has been shown in previous cross-country skiing sprint studies (Andersson et al., 2010; Sandbakk et al., 2011), downhill section times were not related to total race time in the present study. Nevertheless, downhill performance might affect competition results when the margins between winning and losing are small. For example, in the present study the fastest athlete was 0.9 s faster in the final downhill section compared to the second-fastest athlete, while the winning margin in total race time was only 0.8 s. The two downhill sections measured in the present study revealed two distinct and important parts where instantaneous velocities were related to the section times, the first being the velocity during the acceleration phase at the top of the downhill. The velocity at the end of this part corresponded to ~9 m·s^−1^, which is the speed at which skiers have been reported to transfer from active power production into a tucked position (Gløersen et al., 2018b). Therefore, active power production at the beginning of the downhill appears important in order to gain time during the downhill section. The importance of the transition into a downhill might be overlooked by some skiers, resulting in suboptimal power production at this point in the race and a subsequent loss of time over the downhill section. The second important part of the downhill was the turns, in which better section times were related to greater velocities before, during and after the turn. Previously, step turning has been shown to result in higher velocities during all phases of a turn compared to skidding (Sandbakk et al., 2014). In the present study, it is likely that the athletes' turning techniques had an effect on their velocities during the turns and consequently, the downhill section times. Therefore, technique training to attenuate any unnecessary deceleration during downhill turns could be one aspect that could benefit skiers and consequently improve their performance. While the instantaneous velocities reached at the end of the downhill were not related to the overall downhill section times, this end velocity was related to subsequent section time. This suggests that traditional section time analyses might fail to identify some of the important effects that downhill skiing performance has on total performance times.

The findings of this study should be viewed in light of some limitations. Firstly, the sample size was relatively small, with only 11 athletes, resulting in low statistical power. Secondly, the data is only representative of a single classic sprint competition. The generalizability of these findings for different tracks with varying track profiles, race distances and combinations of uphill, downhill, and flat sections remains to be confirmed in future studies. Thirdly, since the measurements took place during a real-world, high-level competition, differences between the athletes' skis and their gliding properties could not be controlled for. This might influence the results, especially for the downhill sections. Lastly, sub-technique identification was not included in the measurements. This means that the observed differences in skiing velocity cannot be associated with the sub-techniques employed. Thus, the combination of SPM and sub-technique identification remains a topic for future studies.

## Practical Applications

The results of the present study are useful for cross-country skiing athletes and coaches to optimize pacing strategies over different track sections, and to identify track locations that are most crucial for performance. Individual results can also be used to identify athletes' strengths and weaknesses at different track locations in order to guide subsequent training interventions and improve overall performance. In future, the statistical parametric mapping method used in the present study could be combined with sub-technique identification to further clarify how skiers achieve higher velocities at crucial track locations.

## Conclusions

The results of the present study have shown that statistical parametric mapping is a useful tool for analyzing cross-country skiing micro-pacing strategies. Micro-pacing strategies were found to relate to overall skiing performance, and distinct uphill and flat track sections were identified where the athletes with faster total race times skied with higher instantaneous velocities compared to the slower athletes. The first major uphill section on the track was characterized by a more conservative micro-pacing strategy compared to the more aggressive micro-pacing strategy used in the last uphill section. Faster times recorded on the flat sections were associated with greater instantaneous velocities throughout those sections. In the downhill sections the acceleration phase and the turns were important in relation to the downhill section times. Based on the results of this study it could be suggested that, in order to improve skiing performance, athletes should focus on more aggressive pacing at various points in a race: (1) early enough during the “end spurt” in order to achieve a fully all-out effort; (2) during the transition from uphill to flat sections, so that acceleration continues after the initial 0–10 m of the flat section; and (3) during the transition from flat or uphill to downhill sections in order to maximize the velocity before transferring into a tucked position. Additionally, skiing faster throughout downhill turns seems to influence downhill section times, so training to improve technique and achieve small time gains in downhill turns may provide an advantage over competitors.

## Data Availability Statement

The datasets generated for this study are available on request to the corresponding author.

## Ethics Statement

This study involving human participants was reviewed and approved by the regional ethical review board of Umeå University, Sweden (#2018-441-32M). The participants provided their written informed consent to participate in this study.

## Author Contributions

SI, EA, and KM contributed to the conception and design of the study. SI and SC performed the statistical analyses. SI wrote the first draft of the manuscript. All authors contributed to manuscript writing and revisions, read, and approved the submitted version.

## Conflict of Interest

The authors declare that the research was conducted in the absence of any commercial or financial relationships that could be construed as a potential conflict of interest.
